# Does Childcare Work Promote Cardiorespiratory Fitness and Health? A Cross-Sectional Study of Danish Childcare Workers Based on Accelerometry and Heart Rate Measurements

**DOI:** 10.3390/ijerph182312496

**Published:** 2021-11-27

**Authors:** Kathrine Greby Schmidt, Rasmus Kildedal, Anders Fritz Lerche, Maja Vilhelmsen, Charlotte Lund Rasmussen, Svend Erik Mathiassen, Leon Straker, Andreas Holtermann

**Affiliations:** 1 The National Research Centre for the Working Environment, 2100 Copenhagen, Denmark; afl@nfa.dk (A.F.L.); mvi@nfa.dk (M.V.); clr@nfa.dk (C.L.R.); aho@nfa.dk (A.H.); 2 Department of Sports Science and Clinical Biomechanics, University of Southern Denmark, 5230 Odense, Denmark; 3 Centre for Musculoskeletal Research, Department of Occupational and Public Health Sciences, University of Gävle, 801 76 Gävle, Sweden; SvendErik.Mathiassen@hig.se; 4 School of Allied Health and enAble Institute, Curtin University, Perth 6102, Australia; L.straker@curtin.edu.au

**Keywords:** childcare workers, Goldilocks Work Principle, physical behaviors, high cardiometabolic intensity, workplace

## Abstract

Childcare workers are reported to have poor cardiorespiratory fitness and health. The Goldilocks Work Principle argues that productive work should be designed with the right composition, intensity and alternations of physical behaviors so that workers get fit and healthy. The purpose of this study was to investigate: (1) composition, (2) intensity and (3) alternations of physical behaviors during work and leisure among childcare workers. Data were collected using accelerometers and heart rate monitors over five workdays among 51 childcare workers at five Danish childcare institutions. Workers mainly spent their work time sedentary (43.0%), spent little time (0.7%) at sufficiently high cardiometabolic intensity to increase cardiorespiratory fitness and often alternated between physical behaviors (67.0% occurred in bouts of <5 min). These findings indicate that the workers have a composition of behaviors at work dominated by sedentary time, little time with high cardiometabolic intensity, and frequent alternations between behaviors. During leisure, workers spent more time sedentary (59.4%), more time at high cardiometabolic intensity (3.4%) and less time occurred in bouts <5 min (38.7%). We see a potential for promoting cardiorespiratory fitness and health of childcare workers by redesigning the way they play with the children, so that work time with high cardiometabolic intensity is increased.

## 1. Introduction

Childcare workers perform an important societal task by providing a safe, healthy and developmentally enhancing environment for children. However, childcare workers are reported to have poor cardiorespiratory fitness and health [1,2,3]. For example, in Denmark childcare workers have lower self-rated health and higher prevalence of musculoskeletal pain compared with the national average across professions [4]. These health issues are barriers to a long and productive working life in childcare [5,6,7].

Workplace interventions have tried to address these health issues by implementing health-promoting workplace interventions in childcare organizations, but with modest or no effect [8,9]. One reason why these interventions, and many other workplace health-promoting initiatives, have failed may be that they have not been an integrated part of the productive work [10]. Thus, these interventions require workers to take time away from their productive work and rely on their motivation to perform additional activity during breaks, limiting its feasibility and sustainability [10]. In contrast, initiatives that target how productive work is organized and performed aiming to promote cardiorespiratory fitness and health among workers may be more feasible and easier to sustain.

The Goldilocks Work Principle has been proposed as a new idea of how to design productive work so it becomes ‘just right’ with respect to the composition, intensity and alternations of physical behaviors (being sedentary, standing and being active) to improve cardiorespiratory fitness and health of workers [11,12]. More specifically, for productive work, it ought to offer, (1) a balanced composition of physical behaviors during the day, (2) sufficient time at cardiometabolic intensities promoting cardiorespiratory fitness, and (3) a sufficient frequency of shifts between physical behaviors to secure variation between physical behaviors and, thus, time for recovery [11].

Thus, to develop workplace interventions in line with the Goldilocks Work Principle, it is necessary to know to what extent the current productive work fulfils these three characteristics [11]. Moreover, in the context of designing health-promoting productive childcare work, it is also valuable to know about the workers’ physical behaviors during leisure. For example, if the workers spend a considerable amount of time with high cardiometabolic intensity at leisure, the need for high cardiometabolic intensity at work may be minimal. Instead, the need for time with low cardiometabolic intensity, allowing recovery, may be more important. On the other hand, if the workers spend much of their leisure time sedentary without high cardiometabolic intensity, a health-promoting design of productive work ought to have less sedentary time and include periods with sufficiently high cardiometabolic intensity [11].

Research on the health of childcare workers is limited [2]. Most occupational studies of childcare workers have investigated traditional ergonomics risk factors such as time spent with forward bent back and kneeling/squatting [13], lifting of children or heavy objects, or time working in awkward postures [14]. Moreover, the limited knowledge available is often based on self-reports, which are known to be imprecise and potentially biased [15]. Only three studies have used accelerometers to investigate childcare workers’ composition of time in physical behaviors during a regular working week [2,13,16]. However, two of these studies did not differentiate between working hours and leisure time [2,16] and the third did not assess leisure time activity at all [13]. Moreover, these studies did not investigate if the childcare workers achieved a sufficiently high cardiometabolic intensity (i.e., at least 60% of the heart rate reserve (HRR)) for a sufficiently long period to increase cardiorespiratory fitness and health, nor did they assess alternations between physical behaviors.

Therefore, the purpose of this study was to investigate, using wearable sensors (i.e., accelerometers and heart rate monitors), the (1) composition, (2) intensity and (3) alternations of physical behaviors during work and leisure among childcare workers.

## 2. Materials and Methods

This paper reports baseline data forming part of the ‘Goldilocks childcare project’, a randomized controlled intervention trial aiming to evaluate the effectiveness of implementing the Goldilocks Work Principle in childcare work [17].

### 2.1. Data Protection and Ethical Approval

The National Research Center for the Working Environment has an institutional agreement with the Danish Data Protection Agency about procedures to treat confidential data (journal number 2015-41-4232), e.g., by securing data at a protected drive with limited access, and by anonymizing all individual data. The Danish National Committee on Biomedical Research Ethics (the local ethical committee of Frederiksberg and Copenhagen) has evaluated a description of this study and concluded that, according to Danish law as defined in Committee Act § 2 and § 1, the intervention described should not be further reported to the local ethics committee (Ref number: H-18041423). The study is registered in the International Standard Registered Clinical/soCial sTudy Number (ISRCTN) registry (ISRCTN15644757).

### 2.2. Recruitment of Childcare Institutions

Childcare institutions were recruited from the greater Copenhagen municipality in collaboration with the employer organizations, unions and local government municipalities. The study was announced through e-mails distributed to the childcare institution managers. In order to be eligible for participation, the institution had to supervise children aged 3–6 years, which is the age at which Danish children attend kindergarten and can actively participate in Goldilocks games [18]. To secure the most efficient use of project resources, a minimum of nine childcare workers employed at each institution was required.

### 2.3. Workers within Participating Centers

Institutions decided whether to participate, but participation in data collection (i.e., the questionnaire, anthropometric and cardiorespiratory fitness testing and accelerometer and heart rate measures) was still voluntary for the individual workers. All workers at the participating institutions were informed about the general aims of the study and those participating gave their written consent. Exclusion criteria were adhesive allergy, fever on the first test day, and pregnancy, since the latter has been shown to influence both heart rate and physical behaviors [19].

### 2.4. Data Collection

**Figure 1:**
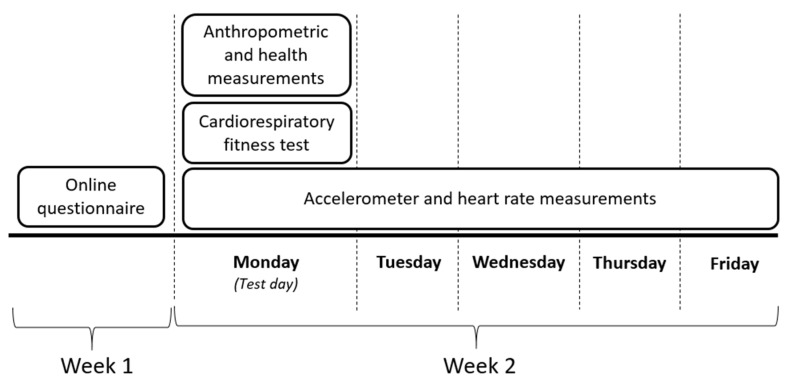
Overview of the data collection process.

Data were collected in 2020 from late January to late February. Figure 1 provides an overview of the data collection process.

#### 2.4.1. Online Questionnaires

Prior to the on-site data collection week, the childcare workers completed an online questionnaire sent in a text message to their mobile phone, using a unique link to the online survey tool SurveyXact (sourced from Ramboll Management Consulting, Aarhus, Denmark). The questionnaire contained questions regarding (1) sociodemographic factors, i.e., age, gender; ethnicity; length of service as a childcare worker and length in current job; job title, (2) health behaviors, i.e., alcohol intake; smoking and (3) self-reported time in moderate to vigorous physical activity (h/week) [20]. Since the questionnaire was distributed by the employer organizations, unions, and local government municipalities, data on the response rate were not available.

#### 2.4.2. Physical Assessment

Anthropometric measurements included height, weight (kg), body mass index (weight (kg)/(height squared (m^2^)), fat percentage (BC-418 MA body composition analyzer; Tanita, Tokyo, Japan) and resting blood pressure (Omron M3 or Omron M6 Comfort; Omron Corporation, Kyoto, Japan). Cardiorespiratory fitness level was assessed using the Ekblom-Bak submaximal cycle ergometer test (Monark AB, Varberg, Sweden) [21], in which the maximal oxygen uptake is estimated based on the difference in heart rate between an initial low standard workload and a subsequent higher ‘final’ workload [22]. During the cycle test, heart rate was monitored using an arm-worn Polar^®^ OH1 heart rate monitor (Polar Electro Oy, Kempele, Finland).

#### 2.4.3. Wearable Sensor Measurements of Physical Behaviors

Physical behavior composition, intensity and alternations were measured using wearable sensors worn continuously over five working days in week 2 (Figure 1). The childcare workers were asked during the measurement days to note in a paper diary what time they (1) woke up, (2) arrived at work, (3) left work, (4) went to sleep, (5) if any of the devices were detached and (6) if they had any sick or leisure days.

An AX3 accelerometer (3-Axis Logging Accelerometer; Axivity Ltd., Newcastle upon Tyne, UK) was mounted using adhesive tape (Hair-Set double-sided adhesive tape; 3 M Company, Maplewood, MN, USA) on the childcare worker’s right thigh at the most prominent part of the quadriceps femoris, midway on the line between the anterior inferior iliac spine and the upper edge of the patella. Additionally, a FirstBeat Bodyguard 2 heart rate monitor (FirstBeat Technologies Ltd., Jyväskylä, Finland) was mounted with Ag/AgCl pre-gelled electrodes (Ambu WhiteSensor CMM00-S/30; Ambu A/S, Ballerup, Denmark) below the right clavicle and at the left ribcage.

The diaries were digitized and used to identify working hours, leisure time and sleep periods for accelerometer and heart rate data.

A custom-made MatLab-based program (Acti4; The National Research Centre for the Working Environment, Copenhagen, Denmark) was used to identify physical behaviors from the accelerometer recordings, i.e., sitting/lying, standing, moving, walking, running, stair climbing, cycling and rowing. The last five behaviors were collapsed into the category ‘active’. The program has a documented high sensitivity and specificity [23]. Sleeping periods were determined on the basis of the self-reported sleeping hours in the diary and adjusted if no movement was detected for more than 5 min after the workers had noted they got up from bed. Sick days and leisure days were excluded from analysis. Workdays were excluded from analysis if they comprised less than 4 h of accelerometer measurements [24]. For each participant the average time at work per day was calculated along with leisure time (combined before work hours and after work hours).

The heart rate data were downloaded using the FirstBeat Uploader software (FirstBeat Uploader Version 3.1.2.0; FirstBeat Technologies Ltd., Jyväskylä, Finland) and further processed for evaluation of beat errors using an established software [23,25]. Relative heart rate was expressed in percent of the heart rate reserve (% HRR), i.e., the difference between the estimated maximal heart rate (HRmax = 208 − 0.7 × Age) [26] and the resting heart rate. Resting heart rate was defined as the lowest recorded heart rate value (average of 10 heart beats) during the first night’s sleep. Workdays were excluded from analysis if more than 50% of the heart rate measurements were missing [27].

### 2.5. Statistical Analyses

All data from the questionnaires and the physical assessments were imported into SPSS (IMB SPSS Statistics for Windows, version 24.0.0.; IBM Corp: Armonk, NY, USA). Following quality control and visual confirmation of data distributions not deviating from normality, descriptive statistics were calculated for anthropometric variables, cardiorespiratory fitness and physical behavior variables for each period (i.e., work and leisure), and summarized in terms of means (absolute values and percentages) and standard deviations between workers.

Accelerometer and heart rate data were analyzed to characterize the composition, intensity and alternations of physical behaviors using Rstudio (RStudio Team (2020). RStudio: Integrated Development for R. RStudio, PBC, Boston, MA, USA) and Microsoft Excel (2016). Composition was expressed as time in physical behaviors classified in four categories; (1) sedentary, i.e., sitting and lying; (2) standing, i.e., stationary standing and standing with movement; (3) active, i.e., walking, running, stair climbing, cycling and rowing; (4) sleeping (i.e., the period between ‘went to sleep’ and ‘woke up’). The proportion of time in the first three physical behaviors during work, leisure and in total time awake was calculated relative to the total duration of the period assessed (e.g., work time). Intensity was expressed as the time at low, medium and high cardiometabolic intensities, classified as ≤25% HRR, >25% HRR–<60% HRR and ≥60% HRR, respectively. The proportion of time in each %HRR class during each period (work, leisure, and time awake) was calculated relative to the total duration of heart rate recordings during that period.

To assess alternation between physical behaviors (i.e., sedentary, standing and active), time spent in bouts of <5 min, 5–30 min, and >30 min was calculated for each of the three behaviors. Results are presented in a modified Exposure Variation Analysis (EVA) [28]. This was performed separately for work, leisure and time awake. In this behavior-by-bout duration matrix, time was expressed in minutes per day and as a percentage of the overall work, leisure and awake times, respectively.

Finally, we calculated correlations for accelerometer and heart rate data between work and leisure, in order to examine the extent to which behaviors and intensities in the two periods may be influenced by each other.

## 3. Results

### 3.1. Participants Flow

**Figure 2:**
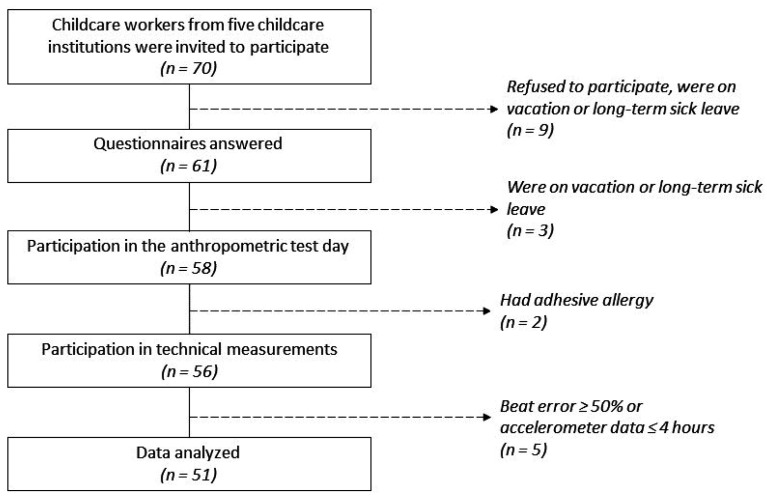
Participant flow, leading to the final sample of 51 childcare workers included in the analysis.

Five Danish childcare institutions agreed to participate and 70 of their workers handling children aged 3–6 years were invited to participate (*n* = 70; Figure 2). Nine of the childcare workers either did not want to participate, were on vacation or on long-term sick leave. Online questionnaires were answered by 61 childcare workers. After answering the online questionnaires, an additional three childcare workers went on vacation or sick leave. The remaining 58 childcare workers all completed the questionnaires and participated in the physical assessments. Two of the 58 childcare workers did not participate in the objective measurement due to adhesive allergy. Five of these fifty-six childcare workers were excluded from analysis due to having either ≤4 h of accelerometer measurements or beat errors ≥ 50% of the heart rate measurements. Fifty-one childcare workers had sufficient data from accelerometer and heart rate measurements and were included in the statistical analysis.

### 3.2. Demographics and Cardiorespiratory Fitness

The childcare workers were predominantly females (55.0%), on average 36.2 (SD 10.3) years old, had an average BMI of 25.0 kg/m^2^ (SD 5.2) and an average blood pressure of 126.5 mmHg (SD 13.0) systolic and 82.6 mmHg (SD 9.5) diastolic (Table 1). On average, the childcare workers reported to engage in moderate to vigorous physical activity 5.3 (SD 4.9) h per week. They had an estimated average maximal oxygen uptake of 44.9 mL/kg/min (SD 12.4).

### 3.3. Composition of Physical Behaviors

A total of 1178 working hours (mean 23.1 h per childcare worker), 1300 leisure time hours (mean 27.1 h per childcare worker) and 1118 sleeping hours (mean 21.9 h per childcare worker) of accelerometer measurements were included in the analysis (Table 2). This corresponded to an average of 3.3 measured days of work per participant and 3.1 measured days of leisure per participant.

The childcare workers spent less time sedentary during work (43.0% of the time awake) compared to leisure (59.4%) and a larger part standing (39.6 vs. 27.9%). Thus, they spent 17.4 and 12.7%, respectively, of their wake time being active at work and during leisure.

### 3.4. Intensity of Physical Behaviors Based on Heart Rate Data

A total of 794 (mean 15.9 per childcare worker) working hours and 1040 (mean 20.8 per childcare worker) leisure time hours (excluding sleep time) of heart rate measurements were included in the analysis (Table 3). This translates to an average of 2.5 measured days of work per participant and 2.7 measured days of leisure per participant.

The childcare workers spent less time ≤25% HRR during work (44.1% of the time awake) compared to leisure (55.1%) and a larger part >25–<60% HRR during work (55.2%) compared to leisure (41.5%). Thus, they spent 15.3 min (3.4%) of their leisure time ≥60% HRR and 2.3 min (0.7%) during work.

### 3.5. Alternation of Physical Behaviors at Work

Table 4 shows the average accumulated time in three bout durations of physical behaviors during working hours and during leisure. During work the largest part of sedentary time occurred in bouts of 5–30 min (95.9 min, 23.1%) and only sedentary behavior occurred in bouts >30 min (28.0 min, 6.7%). The majority of time spent standing and active was in brief bouts of 0–5 min (152.8 min, 36.9%, and 70.4 min, 16.9%, respectively). During leisure, the largest part of sedentary behavior occurred in bouts of >30 min (163.8 min, 31.0%), and workers were more active than at work.

## 4. Discussion

For the childcare workers included in this study, a total of 3678 h of accelerometer measurements and 1834 h of heart rate measurements were analyzed. The findings showed that during working hours, the childcare workers had a composition of physical behaviors dominated by sedentary time (43.0%), and almost no time with high cardiometabolic intensity (0.7%). Most sedentary work time occurred in bouts of 5–30 min (23.1%) and the vast majority of work time spent standing and active was in bouts of <5 min (36.9% and 16.9%). During leisure, workers spent more time sedentary (59.4%), more time at high cardiometabolic intensity (3.4%) and most of their sedentary time occurred in bouts of >30 min (31.0%).

Based on the accelerometer measurements, we found that the childcare workers had a composition of physical behaviors during working hours consisting in 43.0% sedentary, 39.6% standing and 17.4% active. Only one previous study examined these physical behaviors among childcare workers with accelerometer measurements during working hours and found almost identical results (44.8% sedentary, 35.8% standing, 15.5% active) [13]. Other studies have used comparable accelerometer measurements to report on cleaners, manufacturing workers and transportation workers [24] and shift-working nurses [29]. To make a comparison, we combined their results into sedentary, standing and active behaviors and calculated the percentage of time spent on each behavior during an average workday. The amount of sedentary time during working hours (43.0%) of childcare workers was greater than reported for cleaners (24.3%) and manufacturing workers (30.8%) [24], but lower than workers in the transportation industry (62.2%) and shift-working nurses (48.6%) [24,29]. We consider the childcare workers’ exposure to sedentary behavior during working hours to be reasonable to ensure sufficient rest and recovery during the workday, in particular when also considering that most of the time was spent in bouts lasting 5–30 min. Childcare workers were standing (39.6%) more than cleaners (28.6%), but to a similar extent as manufacturing workers (37.2%) [24] and shift-working nurses (38.9%) [29]. Considering that the childcare workers spent a major part of their working hours sedentary, we do not consider the exposure to standing as a health problem. The same applies to the active behaviors, which occurred more (17.4%) than among shift-working nurses (12.5%) [29], but less than among cleaners (47.1%), manufacturing workers (32.1%) and workers in the transportation industry (24.3%) [24]. Referring to guidelines from the European Agency for Safety and Health at Work [30], we consider the childcare workers’ composition of physical behaviors during working hours to be healthy. That said, there is a lack of evidence in the literature regarding the health effects of compositions of physical behaviors among childcare workers. Thus, we recommend future studies to improve knowledge in this area.

Based on the heart rate measurement, we found that the childcare workers spent hardly any of their working hours at a high cardiometabolic intensity (<1% time at ≥60% HRR, corresponding to about 2.3 min in a 6.9 h workday). This result is in agreement with a small proof-of-concept study (*n* = 19) finding that childcare workers spent <1% of their work time at a high cardiometabolic intensity [18]. Moreover, the proof-of-concept study found that it was feasible to redesign a core work task—playing with the children—in childcare according to the Goldilocks Work Principle, leading to a substantial increase in time with high cardiometabolic intensity among the childcare workers. Considering that the childcare workers in the current study had almost no time at a high cardiometabolic intensity during work, and that time spent at high cardiometabolic intensity has been shown to improve cardiovascular fitness [31,32], further studies should examine initiatives for designing productive childcare work so that childcare workers obtain more work time with high cardiometabolic intensity and thereby increase their cardiorespiratory fitness and health.

At work, sedentary behavior mostly occurred in bouts of 5–30 min, while time spent standing and active mainly occurred in shorter bouts of 0–5 min. Overall 67.0% of physical behaviors occurred in bouts of <5 min and so it appears that the childcare workers often alternate between physical behaviors, especially when standing and being active. We have not been able to compare this to previous studies, since, to our knowledge, this is the first study to report alternations of physical behaviors during childcare work. Further, knowledge on the ‘just right’ frequency of alternations between physical behaviors is lacking. However, prolonged bouts of sedentary behavior or standing at work have been found to be associated with poor health [33,34,35,36]. Since no prolonged bouts of standing occurred during work, and very little work time was spent in prolonged bouts of sedentary behavior (6.7%), there seems to be limited potential for health benefits by increasing alternations between physical behaviors during childcare work.

It is desirable to take childcare workers’ leisure time behaviors into account when assessing whether it would benefit workers’ cardiorespiratory fitness and health to be more active with high intensity at work. We found that the leisure time composition of the childcare workers was dominated by sedentary time (59.4%), mainly occurring in long bouts, i.e., >30 min (31%). Thus, the childcare workers spent considerable time being sedentary, seen over the whole day (43.0% at work and 59.4% at leisure), which might increase their risk of negative effects, including cardiovascular disorders and reduced physical capacity [37,38]. On the other hand, a balance of relaxation and exercise after work may be important to help manage work stress.

Moreover, we found that the childcare workers spent more time at high cardiometabolic intensity during leisure (3.4% time at ≥60% HRR, i.e., about 15.3 min) than during work (0.7% time, 2.3 min). We expected this, since leisure time physical activity often includes dynamic movements at higher intensity levels, such as deliberate training activities [39]. We did not find any major correlations between time spent on physical behaviors during work and leisure (Appendix A), and not for the variables reflecting alternations between behaviors either (Appendix C). However, the correlation between times spent at ≤25% HRR during work and leisure was strong, and those between times spent at >25–<60% HRR and ≥60% HRR were moderate (Appendix B). Considering that the correlations between behaviors were small, this is likely a sign that the percentage time at different % HRR categories at work and during leisure is mainly determined by the fitness of the participant.

Although some greater time at a high cardiometabolic intensity was found during leisure than during work, we see a need for—and a great potential in—redesigning childcare work to include more time with high cardiometabolic intensity; one reason being that high-intensity physical activity has been shown to buffer the harmful effect on health from extensive sedentary time [40]. Moreover, more time at work with a high cardiometabolic intensity would increase the cardiorespiratory fitness of most childcare workers, which is known to be a strong protective factor against poor health [41].

### 4.1. Practical Implications

By providing novel information about the composition, intensity and alternations of physical behaviors during work and leisure time for childcare workers, our study contributes to the understanding of why the work of childcare workers does not seem to benefit their cardiorespiratory fitness and health, and also which interventions might be needed to redesign their work so it can promote health. We have previously shown that redesigning the way childcare workers play with the children can lead to more time at a high cardiometabolic intensity [18]. Therefore, we plan to conduct a randomized controlled trial investigating if active play with the children improves cardiorespiratory fitness and health of childcare workers.

### 4.2. Strengths and Limitations

A strength of our study was the high worker participation rate (82%). Another strength was the use of validated accelerometer measurements [23] to capture physical behaviors over multiple workdays, and the use of heart rate recordings to estimate the relative cardiometabolic intensity of each individual worker [42]. A final strength of our study was the use of a diary to differentiate between the domains work, leisure and time in bed at night.

However, a limitation was the exclusion of some heart rate data due to a beat error ≥ 50%. A high beat error could be due to rapid body movements (e.g., during high intensity physical behaviors) disturbing the signal, and therefore time spent at ≥60% HRR may have been higher than reported in this study. Another limitation was that we only measured leisure time during working days, and not during non-workdays and weekend days. Thus, the reported physical behaviors during leisure time might not be representative of the total weekly leisure time. However, physical activity levels per day on week days and weekend days has previously been reported for 553 childcare workers [8], indicating that the childcare workers are less active during the weekend days. Future studies of childcare workers should include wearable sensor measurements even during non-workdays and weekend days. A final potential limitation is selection bias of participating childcare institutions. The childcare institutions were recruited only from the greater Copenhagen municipality, which might not be representative for the whole of Denmark.

## 5. Conclusions

This study gives a novel contribution to our understanding of the composition, intensity and alternations of physical behaviors among childcare workers during both work and leisure. The results indicate that childcare work is composed of behaviors dominated by sedentary time, very little time with high cardiometabolic intensity and frequent alternations between behaviors. During leisure we found that the workers spent more time sedentary, more time with high cardiometabolic intensity and more time in long sedentary bouts. Overall, the composition and the alternations of physical behaviors during childcare work appear healthy. However, the time spent with high cardiometabolic intensity is not sufficient to increase the cardiorespiratory fitness and health of the workers. Thus, we see a potential in promoting cardiorespiratory fitness and health among childcare workers by increasing their work time at a high cardiometabolic intensity, preferably by redesigning the way they play with the children. Further studies should investigate the effectiveness of implementing such a change in childcare work in improving cardiorespiratory fitness and health among childcare workers.

## Figures and Tables

**Table 1:** Demographic, anthropometric and health characteristics and cardiorespiratory fitness level of the childcare workers (*n* = 51). Values are presented as frequencies (*N*), percentages (%), and mean (with SD).

Variables	*N* (%)	Mean (SD)
Gender (female)	28 (55.0)	
Age (years)		36.2 (10.3)
Born in Denmark	44 (86.3)	
Length of service in current job (years)		3.6 (1.2)
Length of service in current profession (years)		10.3 (7.6)
Job title		
Childcare worker	28 (54.9)	
Childcare assistant	20 (39.2)	
Other	3 (5.9)	
Average alcohol intake (standard drinks/week)		1.8 (0.7)
Current smokers (yes)	12 (23.5)	
Self-reported time in moderate to vigorous physical activity (h/week)		5.3 (4.9)
Blood pressure (mmHg)		
Systolic		126.5 (13.0)
Diastolic		82.6 (9.5)
BMI (kg/m^2^)		25.0 (5.2)
Maximal oxygen uptake (mL/kg∙min) *		44.9 (12.4)
Female		37.6 (8.7)
Male		53.7 (10.1)

* *n* = 50.

**Table 2:** Accelerometer-based time spent during a working day among the 51 childcare workers in four categories of physical behaviors, i.e., sedentary (i.e., sitting and lying), standing (i.e., stationary standing and standing with movement), being active (i.e., walking, running, cycling, stair climbing, rowing) and sleep. Results are presented as minutes (mean, SD) and percentages (% of time awake, SD) of time at work, during leisure and for the total time awake.

Behaviors	Work Time		Leisure Time		Awake Time		Sleep Time
	Minutes (SD)	% (SD)	Minutes (SD)	% (SD)	Minutes (SD)	% (SD)	Minutes (SD)
Sedentary	178.1 (51.2)	43.0 (10.5)	310.6 (76.5)	59.4 (11.5)	488.7 (91.3)	52.2 (7.1)	-
Standing	163.7 (40.0)	39.6 (8.0)	144.0 (52.7)	27.9 (9.1)	307.7 (63.6)	33.0 (5.6)	-
Active	72.1 (20.8)	17.4 (4.1)	66.8 (28.4)	12.7 (4.5)	138.9(36.0)	14.8 (3.3)	-
Sleep	-	-	-	-	-	-	459.0 (64.4)

**Table 3:** Minutes spent at ≤25, >25–<60 and ≥60% heart rate reserve during an average working day, including leisure time, but excluding sleep. Results are presented as minutes (mean, SD) and percentages (% of time awake, SD) of time at work during leisure, and for the total time awake.

Intensity	Work Time		Leisure Time		Awake Time	
	Minutes (SD)	% (SD)	Minutes (SD)	% (SD)	Minutes (SD)	% (SD)
≤25% HRR	159.2 (91.4)	44.1 (25.7)	254.2 (97.9)	55.1 (20.0)	413.3 (175.6)	50.1 (20.8)
>25–<60% HRR	198.2 (92.1)	55.2 (25.3)	191.6 (91.0)	41.5 (18.9)	389.7 (162.2)	47.7 (20.2)
≥60% HRR	2.3 (3.4)	0.7 (1.0)	15.3 (13.0)	3.4 (3.1)	17.6 (14.8)	2.2 (2.0)

**Table 4:** Exposure variation analysis (EVA) of physical behaviors during work, distributed into nine categories combining three behavior types, i.e., sedentary, standing and active, and three different bout lengths, i.e., <5, 5–30 and >30 min. Results are presented as minutes (mean, SD) and percentages (% of time awake, SD) of time at work, during leisure and for the total time awake.

Behavior by Bout	Work Time		Leisure Time		Awake Time	
	Minutes (SD)	% (SD)	Minutes (SD)	% (SD)	Minutes (SD)	% (SD)
Sedentary < 5 min	54.2 (17.7)	13.1 (3.9)	32.7 (13.1)	6.6 (3.8)	86.9 (24.1)	9.4 (2.8)
Sedentary 5–30 min	95.9 (32.4)	23.1 (7.3)	114.2 (42.2)	21.7 (7.9)	210.1 (50.8)	22.4 (4.9)
Sedentary > 30 min	28.0 (37.0)	6.7 (8.4)	163.8 (76.4)	31.0 (14.0)	191.8 (90.2)	20.3 (9.1)
Standing < 5 min	152.8 (35.6)	36.9 (7.2)	112.9 (38.5)	22.0 (7.4)	265.7 (48.7)	28.5 (4.5)
Standing 5–30 min	11.9 (9.1)	2.8 (2.1)	30.4 (24.7)	5.7 (4.5)	42.2 (26.5)	4.5 (2.7)
Standing > 30 min	0.2 (1.1)	0.0 (0.2)	1.3 (5.4)	0.2 (1.1)	1.4 (5.5)	0.2 (0.6)
Active < 5 min	70.4 (20.0)	16.9 (4.0)	52.8 (18.3)	10.1 (3.1)	123.1 (27.9)	13.2 (2.6)
Active 5–30 min	1.9 (3.4)	0.4 (0.8)	12.8 (14.7)	2.4 (2.6)	14.6 (15.7)	1.5 (1.6)
Active > 30 min	0.0 (0.0)	0.0 (0.0)	1.4 (6.2)	0.2 (1.0)	1.4 (6.2)	0.1 (0.6)

## Data Availability

The data presented in this study are available on request from the corresponding author. The data are not publicly available due to general data protection regulations (GDPR), and personal data ordinance (PDPO).

## Appendix A

**Table A1:** Pearson correlation coefficients between time in different physical behaviors (minutes) at work and during leisure (*n* = 51).

	Sedentary Work Time	Standing Work Time	Active Work Time
Sedentary Leisure Time	−0.02	0.10	0.06
Standing Leisure Time	0.16	−0.08	−0.22
Active Leisure Time	0.09	−0.03	0.05

## Appendix B

**Table A2:** Pearson correlation coefficients between time in different intensities (% HRR, minutes) at work and during leisure (*n* = 51).

	≤25% HRR Work Time	>25–<60% HRR Work Time	≥60% HRR Work Time
≤25% HRR Leisure Time	0.72	−0.49	−0.30
>25–<60% HRR Leisure Time	−0.57	0.57	0.07
≥60% HRR Leisure Time	−0.18	0.13	0.44

## Appendix C

**Table A3:** Pearson correlation coefficients between time in work and leisure behaviors (minutes) stratified by bout length (*n* = 51).

	Sedentary < 5 min Work Time	Sedentary 5–30 min Work Time	Sedentary > 30 min Work Time	Standing < 5 min Work Time	Standing 5–30 min Work Time	Standing > 30 min Work Time	Active < 5 min Work Time	Active 5–30 min Work Time	Active > 30 min Work Time
Sedentary <5 min Leisure Time	0.20	0.14	0.04	−0.31	−0.25	−0.09	−0.23	−0.03	NA
Sedentary 5–30 min Leisure Time	0.20	0.14	0.04	−0.31	−0.25	−0.09	−0.23	−0.41	NA
Sedentary > 30 min Leisure Time	−0.18	−0.11	0.16	0.06	0.17	0.19	0.05	0.09	NA
Standing < 5 min Leisure Time	0.11	0.15	0.07	−0.14	−0.07	−0.17	−0.11	−0.13	NA
Standing 5–30 min Leisure Time	−0.05	0.24	−0.08	0.05	0.03	0.01	−0.28	0.11	NA
Standing > 30 min Leisure Time	−0.11	0.07	−0.14	0.05	−0.03	−0.03	−0.05	−0.06	NA
Active < 5 min Leisure Time	0.14	0.20	0.06	−0.03	−0.17	−0.21	0.06	−0.13	NA
Active 5–30 min Leisure Time	0.04	0.00	−0.14	0.09	−0.14	−0.03	0.01	0.20	NA
Active > 30 min Leisure Time	−0.11	−0.18	0.19	0.01	−0.19	−0.03	0.02	0.00	NA
